# Epidermolytic hyperkeratosis of the vulva: Case report and review of the literature

**DOI:** 10.1002/ski2.325

**Published:** 2023-12-27

**Authors:** Dilshad Sachedina, Camila Villa‐Ruiz, Candice Brem, Debjani Sahni

**Affiliations:** ^1^ Department of Dermatology Boston Medical Center Boston Massachusetts USA; ^2^ Section of Dermatopathology Department of Dermatology Boston University Chobanian & Avedisian School of Medicine Boston Massachusetts USA

## Abstract

Epidermolytic hyperkeratosis is a rare histopathological phenomenon which has been reported in a number of dermatological conditions. It is rare but can cause chronic and intractable symptoms which can impede the quality of life of those affected. Treatment options are variable and not enough data exists to provide a definitive protocol for management. We present this case to highlight a simple, efficacious way for dermatologists to treat the condition and provide a literature review.

## Graphical abstract

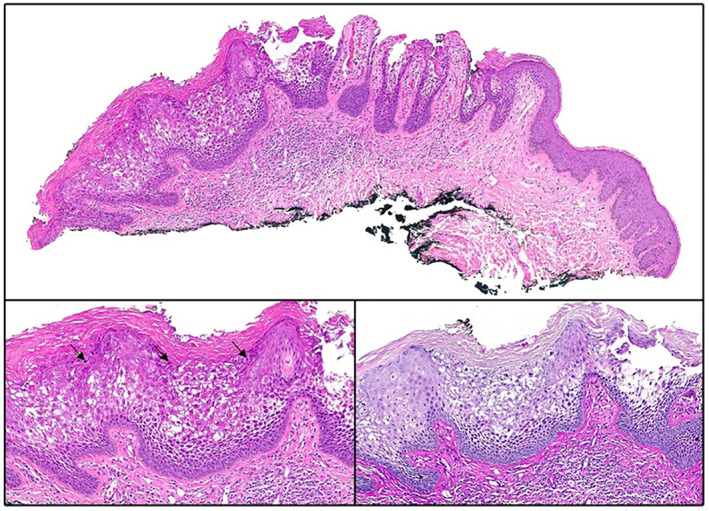

## 1 INTRODUCTION

Epidermolytic hyperkeratosis (EHK) is a rare histopathological phenomenon which has been reported in a number of dermatological conditions. It is rare but can cause chronic and intractable symptoms which can impede the quality of life of those affected. Treatment options are variable and not enough data exists to provide a definitive protocol for management. We present this case to highlight a simple, efficacious way for dermatologists to treat the condition and provide a literature review.

## 2 CASE SYNOPSIS

**FIGURE 1:**
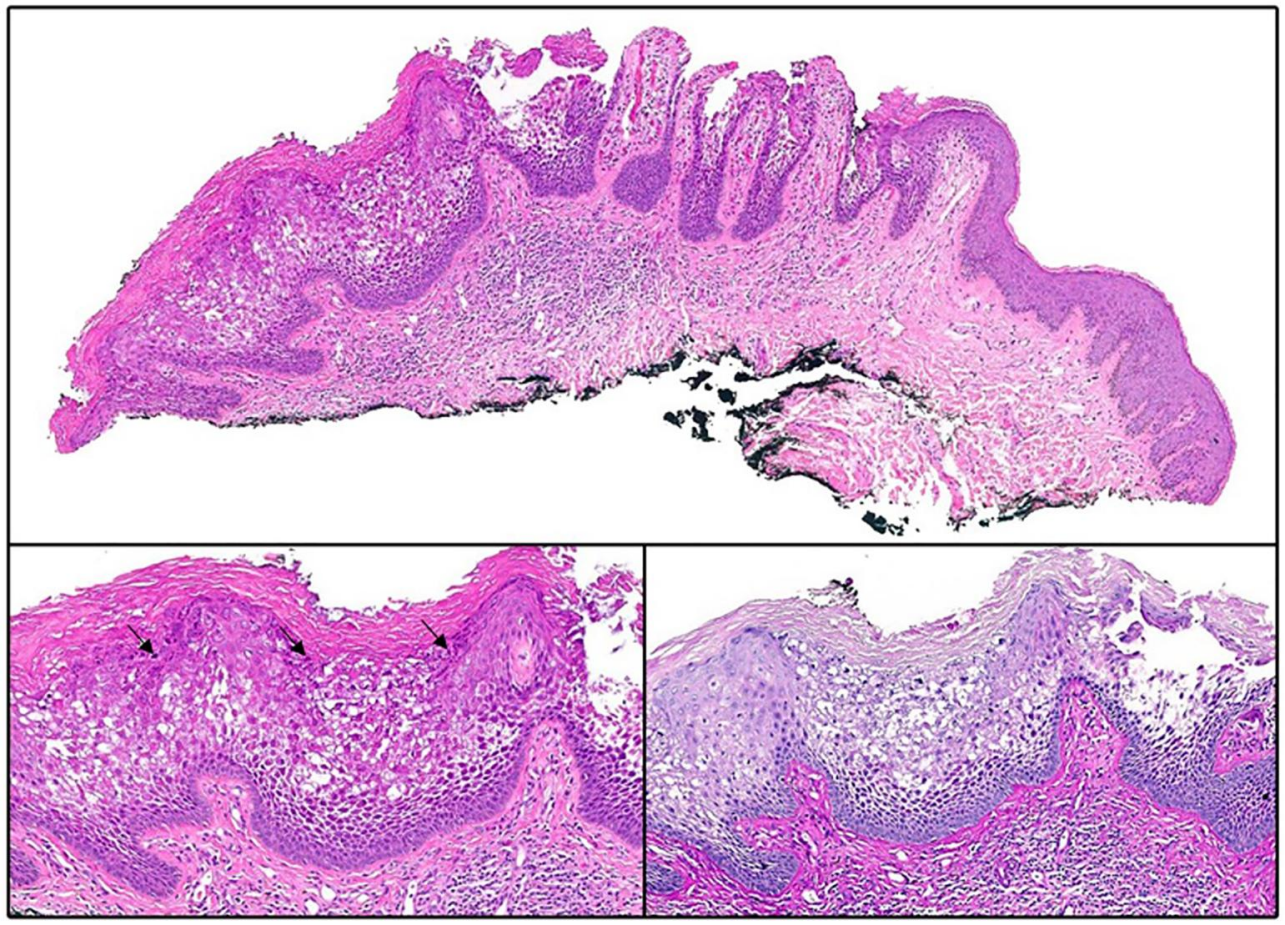
Top: low power examination reveals hyperkeratosis and mild epidermal hyperplasia with slight epidermal pallor and hypergranulosis (H&E, 40×). Bottom left: higher power inspection reveals both coarse keratohyalin granules (black arrow) and variable vacuolar degeneration of the granular and spinous layers (H&E, 100×). A PAS stain was negative for fungal hyphae (PAS, 100×). PAS, Periodic acid–Schiff.

A 67‐year‐old female with no prior dermatological history was referred to gynaecology for a vulvar rash. She had reported longstanding pruritus and genital lesions spanning 2 decades that interfered with her quality of life. Her pruritus was quantified using the verbal rating scale (VRS) at a 4, denoting very severe pruritus. There was no associated vaginal discharge. A shave biopsy, taken instead of a punch biopsy due to the patient's reluctance to have any sutures, from the vulva revealed epidermal hyperplasia demonstrating hypergranulosis and superficial to mid epidermal vacuolar degeneration (Figure 1, top and bottom left). A Periodic acid–Schiff stain was negative for fungal hyphae (Figure 1, bottom right), and there was no evidence of malignancy. These changes were consistent with the EHK reaction pattern. Given the anatomic location and clinical setting, EHK of the vulva was favoured.

The patient was referred to dermatology for further management of her persistent symptoms. Physical examination revealed approximately 30 clustered, grey to white coloured, flat‐topped, shiny monomorphic papules with no papulovesicular or scaly component. Differential diagnoses considered included lichen planus, condyloma acuminata, chronic spongiotic dermatitis, and vaginal inclusion cysts, however pathology results did not demonstrate features consistent with these. The patient was prescribed augmented betamethasone 0.05% ointment BID for 2 months. Within 1 month of twice daily application of the steroid, the patient reported complete resolution of her pruritus for the first time in 30 years. On clinical exam there were only a few remaining scattered vulval papules, with the majority of prior lesions having resolved completely. Her pruritus VRS score came down to 0, denoting no itch. The patient currently uses short bursts of the betamethasone ointment for a couple of days at a time to control any recurrence of her symptoms and has remained well controlled on this regimen for 1 year to date.

## 3 CASE DISCUSSION

EHK is now considered to be a histologic reaction pattern.^1^ While originally EHK was associated with bullous congenital ichthyosiform erythroderma,^2^ focal EHK has recently been described within seborrhoeic keratoses, epidermal nevi and verrucae.^3^ Additionally, solitary lesions demonstrating EHK in the vulval and scrotal regions, termed epidermolytic acanthoma, have also been documented.^4^, ^5^ EHK of the vulva is exceedingly rare. In one study that aimed to demonstrate the relative frequency of various gynaecological dermatoses, EHK made up just one of 183 total cases.^6^

From the literature review we performed, the average age of presentation for EHK of the vulva was 53.4 years old and the average time taken from first presentation to diagnosis was 8.9 years (Table 1).

**TABLE 1:** Previously described cases of epidermolytic hyperkeratosis of the vulva.

References	Age at presentation	Clinical features	Time to diagnosis	Treatment
Iglesias‐Plaza et al.^1^	61	Well circumscribed white hyperkeratotic plaque measuring 10 mm across inner aspect of the left labium majus	2 months	Patient opted not to have any treatment
High et al.^2^	54	Multiple asymptomatic slightly white appearing papules on external vulva	20 years (at least one of the lesions)	Patient did not want treatment once she found out it was benign
Dai et al.^3^	68	Multiple, discrete, whitish, well defined, sessile papules with confluence into small plaques localised to the R labia majora	Several years (not quantified)	Refractory to high potency topical steroids and topical oestrogen
Russell et al.^7^	69	Itching and burning of her vulval skin. Vulval and vaginal inflammation. Discrete pale, pearly, warty lesions, each measuring 3–4 mm across and 2–3 mm high.	6 weeks	Symptoms improved with 0.1% oestriol. 2% miconazole and 1% hydrocortisone
Wesley‐Fletcher et al.^8^	59	4 mm verrucous papule noted on the L labium majorum surrounded by smaller other 1–2 mm hyperkeratotic papules	Several months	Softening with a topical lactic acid preparation
Thomas et al.^9^	50	Hyperpigmented, linear, greyish‐white plaque involving the outer aspect of the labia majora on the left side	Approximately 30 years	Rx not described
Swann et al.^10^	58	Seven scattered, tan to brown, verrucoid papules on the labia and mons pubis resembling condyloma accuminata or Bowenoid papulosis	2 years	Not discussed for the specific patient
Quinn et al.^11^	75	3 mm × 2 mm × 2 mm vulvar papule that was excised	1 year	Not discussed
	40	White hyperkeratotic plaque on the vaginal wall in addition to white plaques on the oral mucosa	Unknown	Not discussed

Lesions of EHK of the vulva can present unilaterally or bilaterally and are typically described as white hyperkeratotic papules that can form confluent small plaques.^1^, ^2^, ^3^, ^7^ In most cases, the presenting complaint is intense pruritus, though some women are asymptomatic whose lesions are incidentally noted by their healthcare provider during routine clinical examinations. The exact aetiology of these lesions is unclear, although a rare case of epidermolytic acanthoma of the scrotum has been thought to be triggered by chronic traumatisation (i.e., itching and scratching),^4^ and there is no documentation in the literature of malignant transformation of either epidermolytic acanthoma or EHK of the vulva. Patients can therefore be reassured of a benign inflammatory process based on the biopsy results.

Treatment options, so far, have focused on a combination of conservative measures such as emollients and mild keratolytic agents including lactic acid, salicylic acid, and glycolic acid.^1^, ^8^ Occasionally, locally destructive therapies such as liquid nitrogen, curettage, and electrodessication and cautery have been used; however, these are painful, potentially disfiguring treatments that may cause scarring and resultant unwanted complications.^2^ The cutaneous eruptions can be refractory to high potency topical steroid and topical oestrogen.^3^ We can only speculate that oestrogen was prescribed in this case for a presumed atrophic vaginitis related pruritus rather than for targeted treatment of any visible hyperkeratotic disease. In one case^7^ a patient with a clinical and histopathological diagnosis of EHK had significant improvement with a combination of 0.1% oestriol, 2% miconazole and 1% hydrocortisone creams, though it is hard to delineate exactly which of these provided the beneficial effect.

Topical calcineurin inhibitors are not yet mentioned in the literature as a treatment option. One case report noted complete resolution of scrotal epidermolytic acanthomas following a 4‐week course of 5% imiquimod ointment,^12^ but the potentially painful, inflammatory, and systemic side effects caused by use of imiquimod on sensitive areas of the body must be taken into consideration.

## 4 CONCLUSION

This is the first case of EHK of the vulva in a patient whose disease was successfully controlled with intermittent use of high potency topical steroids. Attaining the correct diagnosis and using potent topical steroids resulted in a significant improvement to her quality of life. We present this case to remind clinicians to consider this rare diagnosis in a patient presenting with pruritic, papular eruptions of the vulva. Additionally, this case highlights how potent topical steroids can bring about intense relief in a rapid and effective manner in symptomatic patients who have suffered with years of discomfort.

While there have been reports in the literature of EHK becoming quiescent and not progressing even without topical treatment,^2^ a significant proportion of patients struggle with debilitating pruritus for up to 30 years.^9^ Our recommendation in symptomatic individuals is to use high potency topical steroid continuously for a prolonged period (1–2 months), similar to the treatment schedule for vulval lichen sclerosis, and then use the steroid intermittently in shorter burst for maintenance of disease control.

These findings are of heightened importance given the recently recognized dearth of literature surrounding women's health. The condition affects women from menarche to menopause and based on our literature review, a significant proportion of women were misdiagnosed and suffered years of debilitating pruritus prior to biopsies being taken. The appearance of pruritic whitish‐grey, sessile, verrucous papules should alert primary care physicians, gynaecologists, and dermatologists to the possibility of this diagnosis and need for biopsy.

## CONFLICT OF INTEREST STATEMENT

The authors declare no conflicts of interest.

## AUTHOR CONTRIBUTIONS

**Dilshad Sachedina**: Writing—original draft (equal). **Camila Villa‐Ruiz**: Conceptualization (equal); writing—review and editing (equal). **Candice Brem**: Writing—review and editing (equal). **Debjani Sahni**: Conceptualization (equal); writing—review and editing (equal).

## ETHICS STATEMENT

Consent for the publication of all patient photographs and medical information was provided by the authors at the time of article submission to the journal, stating that all patients gave consent for their photographs and medical information to be published in print and online and with the understanding that this information may be publicly available.

## Data Availability

The data that support the findings of this study are available from the corresponding author upon reasonable request.
